# Skin microbiome engineering: Challenges and opportunities in skin diseases treatment

**DOI:** 10.1002/imo2.70012

**Published:** 2025-03-28

**Authors:** Yiang Lyu, Juntao Shen, You Che, Lei Dai

**Affiliations:** ^1^ CAS Key Laboratory of Quantitative Engineering Biology, Shenzhen Institute of Synthetic Biology, Shenzhen Institutes of Advanced Technology Chinese Academy of Sciences Shenzhen China; ^2^ HKU‐Pasteur Research Pole, School of Public Health, Li Ka Shing Faculty of Medicine The University of Hong Kong China; ^3^ University of Chinese Academy of Sciences Beijing China

**Keywords:** immune modulation, microbiome engineering, skin barrier, targeted therapies

## Abstract

The skin microbiome, consisting of a vast array of microorganisms, is essential for human skin health, aiding in barrier protection, immune regulation, wound repair, and defense against pathogens. Disruptions in this microbial balance are closely linked to the onset and worsening of various skin disorders. This review evaluates the potential of skin microbiome engineering as a therapeutic strategy for treating skin diseases. We discuss nontargeted approaches like probiotics and fecal microbiota transplantation that aim to reshape the microbial community, as well as targeted methods such as phage therapy, phage lysins, and engineered bacteria, which specifically modulate microbial populations or influence the skin environment. These approaches open new avenues for personalized dermatological treatments. Despite significant progress, challenges remain in the clinical translation of microbiome‐based therapies. Safety, standardization, regulatory approval, and long‐term ecological stability must be addressed to ensure efficacy and reproducibility in clinical settings, underscoring the critical need for further research in their dermatological applications.

## Highlights

- The skin microbiome is essential for skin health, playing a key role in barrier integrity, immune regulation, and pathogen defense.
- Microbiome engineering offers promising therapeutic strategies, including probiotics, microbiome transplantation, phage therapy, and engineered bacteria.
- Challenges remain in clinical translation, particularly in standardization, safety assessment, and the integration of microbiome‐based therapies into precision medicine.

## Graphical abstract

The skin microbiome plays a crucial role in skin health, influencing barrier integrity, immune responses, and disease susceptibility. Various interventions can reshape the microbiome, broadly categorized into targeted and untargeted approaches. Targeted strategies, such as phage therapy, engineered bacteria, and phage lysins, selectively modulate specific microbial populations, restoring balance and mitigating pathogenic influences. Untargeted interventions, including probiotics, prebiotics, skin microbiome transplantation, antibiotics, and lifestyle modifications, aim to enhance overall microbial diversity and stability. These strategies hold promise for innovative dermatological therapies, paving the way for personalized microbiome‐based treatments. However, challenges remain in standardization, safety, and long‐term ecological impact, necessitating further research for effective clinical translation.

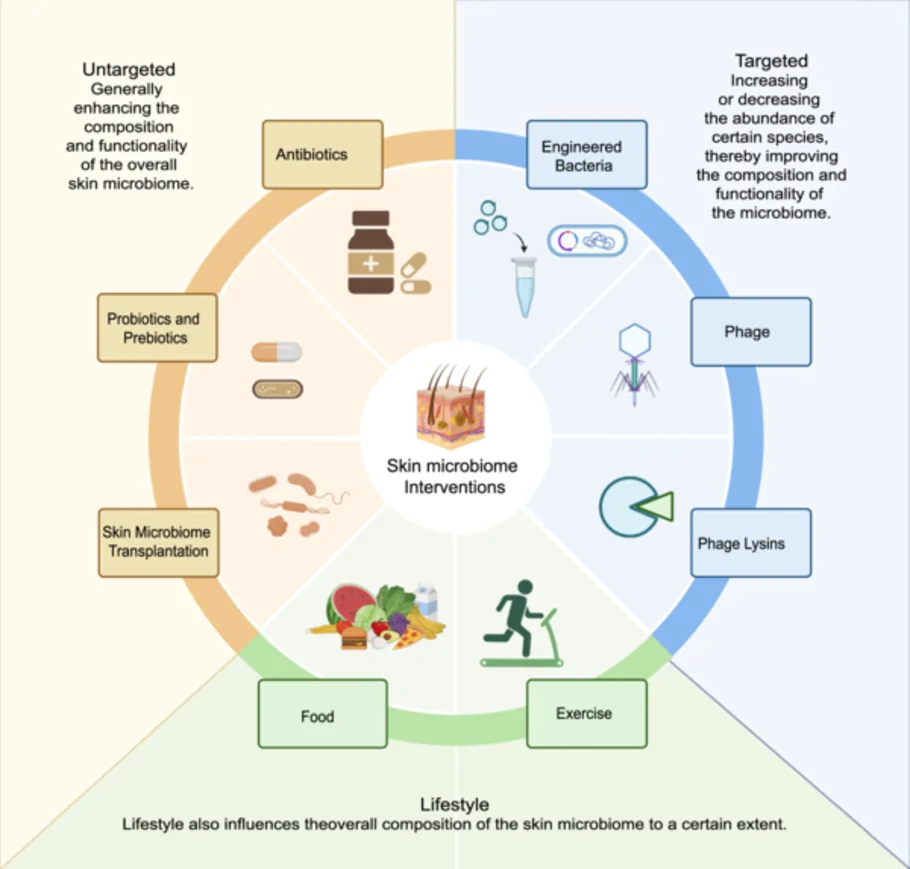

## 1 INTRODUCTION

The skin is the human body's largest organ and consists of three distinct layers: the epidermis, dermis, and subcutaneous tissue [1]. Each layer performs essential physiological functions, forming a robust barrier that protects the body from external threats [2]. The biogeographical diversity of the skin, driven by variations in temperature, humidity, and ion concentration across different sites, supports a wide range of microorganisms [2].

Constantly interacting with the external environment, the skin serves as a dynamic yet relatively stable ecosystem for various microorganisms, including bacteria, fungi, viruses, and archaea. Increasing research has demonstrated that the skin microbiome plays a critical role in maintaining health, influencing dermatological conditions, and impacting skin aging and disease progression [3].

In the past few decades, microbiome research has rapidly advanced, with breakthroughs in microbiome engineering leading to new treatments for conditions such as colitis, Alzheimer's disease, and cancer [4]. Similarly, research into the skin microbiome has highlighted its critical role in skin disease, revealing its involvement in conditions such as acne, eczema, and psoriasis. This review aims to systematically explore the roles of the skin microbiome in health and disease and to compile current methods, case studies, and potential applications of skin microbiome engineering in improving skin health and treating skin conditions. Furthermore, we will address the challenges of developing microbiome‐based therapies and propose future research directions that may revolutionize dermatological treatments and enhance our understanding of skin health.

## 2 ROLE OF THE SKIN MICROBIOME

**FIGURE 1:**
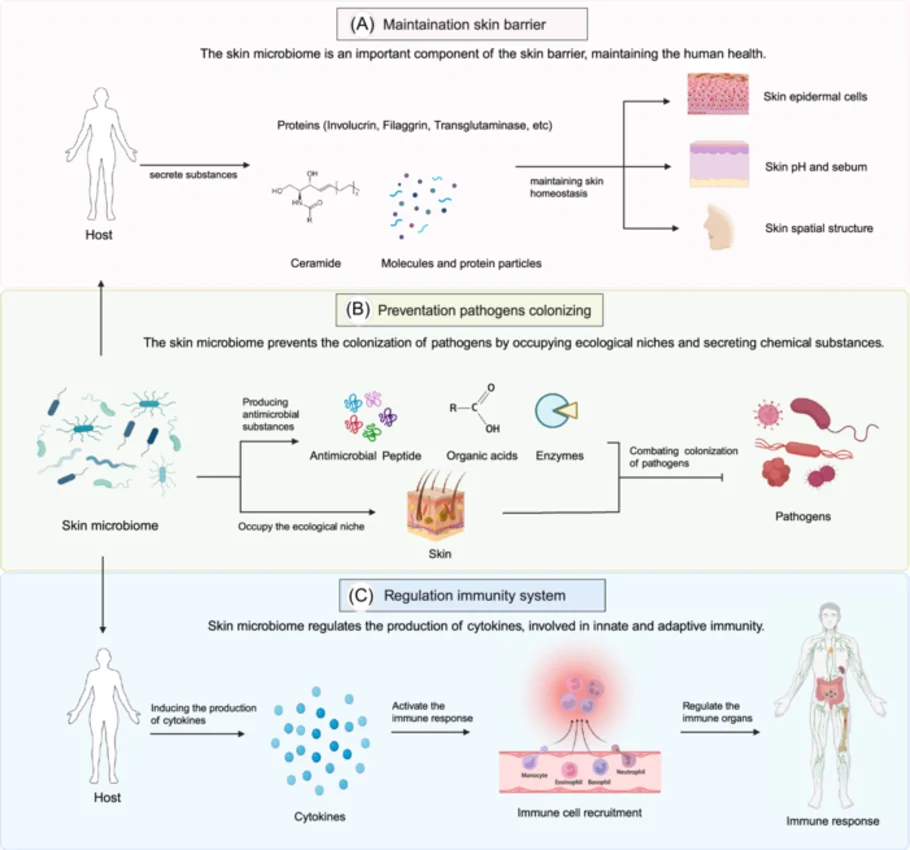
The skin microbiome maintains human health through various mechanisms and pathways. (A) The skin microbiome maintains the homeostasis of skin physicochemical properties by regulating the metabolism of epidermal cells, modulating sebum production and pH balance, and altering the spatial structure between the skin epithelium and skin appendage glands. (B) The skin microbiome prevents the colonization of pathogens by occupying ecological niches and secreting antimicrobial peptides, bacteriocins, organic acids, etc. (C) The skin microbiome activates the innate and adaptive immune systems of the human body by regulating the induction of cytokines to stimulate immune cells.

The skin microbiome and host cells construct the human skin barrier, serving as the first line of defence against the external environment [5]. As a crucial component of the skin barrier, the skin microbiome plays a key role in maintaining human skin health by sustaining physicochemical balance, preventing the colonization of pathogenic microorganisms, and interacting with the host immune system (Figure 1).

### Maintenance of the homeostasis of the physicochemical properties of the skin

The skin is the outermost organ of the human body, and the skin barrier maintains the body's structural integrity, protects other organs and tissues, prevents water loss, and regulates skin pH levels [6]. The physicochemical properties of the skin barrier are not static. Still, they are in a dynamic balance involving continuous aging, regeneration, maturation, and differentiation of epidermal cells, along with ongoing biochemical reactions. The skin microbiome is closely related to the dynamic equilibrium of the skin barrier [7].

The skin microbiome participates in host metabolic processes, producing or consuming certain chemicals to maintain the stability of the skin barrier. Enzymes are central to most biochemical reactions, and evidence suggests that skin microorganisms can secrete various proteases (including filaggrin, involucrin, and transglutaminase) that catalyze the differentiation and maturation of epidermal cells, thereby increasing the rate of skin metabolism and maintaining the homeostasis of the skin barrier [8]. Sebum, including triglycerides, wax esters, and squalene, is an essential component of the skin barrier, primarily synthesized and secreted by sebaceous glands. Ceramides, a type of sebum, are major components of the intercellular spaces of the stratum corneum, constructing the epidermal permeability barrier alongside other lipids [9]. A recent study has found that epidermal *Staphylococcus*, a common skin commensal, can secrete sphingomyelinases that, while providing essential nutrients for itself, produce ceramides utilized by host cells to enhance the integrity and permeability of the skin barrier [10]. Additionally, the lipid film formed by sebum on the skin surface contributes to the slightly acidic environment of the skin [11]. *Cutibacterium acne* (*C. acnes*), another common skin commensal, produces lipases that consume sebum secreted by host cells in the epidermis, altering the composition of the skin lipid layer, producing free fatty acids, and regulating the pH of the skin surface to maintain the acid–base balance of the skin barrier [12]. Furthermore, the skin microbiome can influence the spatial structure of the skin barrier. The attachment of sebaceous glands and sweat glands to the skin is regulated by Claudin (CLDN) proteins [13]. Recent research has shown that the skin microbiome can alter the distance between these attached glands and the epidermis by regulating CLDN expression, thus changing the permeability of the skin barrier [14, 15]. This demonstrates that skin microorganisms can modify the three‐dimensional structure of the skin and spatially regulate the homeostasis of the skin barrier. In summary, the above evidence indicates that the skin microbiome plays a crucial role in regulating skin physiological characteristics.

### Prevention of pathogen colonization

The skin microbiome is crucial in defending against pathogen invasion [16]. Evidence suggests that in the skin microbiome composition of patients with certain skin diseases, the relative abundance of common skin commensals is significantly reduced, which may explain why pathogenic microorganisms are more likely to colonize the skin surface of these patients [17]. In patients with atopic dermatitis (AD), the skin microbiome shows significantly reduced levels of some common commensals like *Streptococcus*, *Cutibacterium*, and *Corynebacterium* compared to healthy individuals [18].

Additionally, skin commensal microorganisms can produce specific chemicals to combat pathogenic microorganisms. Bacteriocins are a class of proteins or peptides produced by bacteria that can kill or inhibit the growth of other bacteria [19]. Studies have found that a variety of bacteriocins can be secreted within the human skin environment, with those produced by coagulase‐negative staphylococci (CoNS), especially *Staphylococcus epidermidis* (*S. epidermidis*), being the most widely distributed. These bacteriocins exhibit significant inhibitory activity against the pathogenic bacterium *Staphylococcus aureus* (*S. aureus*). However, it is important to note that the potential interactions among different microorganisms are often strain‐specific. Even within the same species—*S. epidermidis* or other CoNS strains may produce different types or levels of bacteriocins. Consequently, further investigation into the genetic and functional variations among these strains and how they affect microbial interactions is crucial for understanding the skin microbiome's regulatory mechanisms and developing novel bacteriocin‐based skin therapies [20, 21]. Skin commensal fungi can also produce antimicrobial or antifungal substances to combat pathogen colonization [22]. *Malassezia restricta* and *Malassezia globosa* can produce short‐chain fatty acids (SCFAs) during sebum metabolism, which have multiple pharmacological mechanisms and broad‐spectrum antimicrobial activity against bacteria and fungi [23, 24]. The aspartyl protease 1 (MgSAP1) secreted by *Malassezia globosa* has been shown in vitro to disrupt *S. aureus* biofilm formation by hydrolyzing its protein A [23].

### Regulation and education of the immune system

The human skin microbiome closely interacts with the immune system by regulating responses, maintaining balance, and shaping immune system development. These interactions collectively maintain skin health and the immune system's overall function.

#### Innate immunity

The skin microbiome can participate in innate immune responses in various ways. Cytokines are proteins secreted by the host that carry out specific functions, transmitting signals between cells, regulating and coordinating immune responses, cell proliferation, differentiation, migration, and other biological processes, making them essential components of the innate immune system [25]. The skin microbiome can regulate the production of various including Toll‐like receptors (TLR) [26], interleukin‐1α (IL‐1α) [27], and complement component C5a receptors [28]. Recent evidence suggests this may be related to some anaerobic Gram‐positive cocci on the skin surface [29]. Some skin commensal microorganisms can effectively combat inflammation by upregulating the expression of cytokine‐related genes such as *IL‐8*, *CXCL1*, and *MCP‐1* [30]. The skin microbiome also participates in innate immune responses during wound healing. Skin commensal microorganisms induce host cells to produce cytokine IL‐1β, which is involved in immune responses, thereby accelerating wound healing and skin regeneration [31]. Certain fungi can also trigger unique immune responses in the skin; for instance, *Candida albicans* can stimulate T helper (Th)1/Th17 cells to activate immune responses against skin infections [32].

Skin microorganisms can also induce the host to secrete certain chemicals to enhance its innate immune system. Antimicrobial peptides and proteins (AMPs) are small proteins or peptides secreted by the host that have broad‐spectrum antimicrobial effects and are considered natural antibiotics [33]. Skin commensal *S. epidermidis* and its metabolic products can induce the expression of various AMPs genes, such as *β‐defensin hBD‐2* and *hBD‐3* [34], which effectively combat the colonization of pathogenic *Escherichia coli* (*E. coli*) and *S. aureus*. RNase7, an antimicrobial peptide, has broad‐spectrum antibacterial activity [35]. *Corynebacterium*, an important member of the skin microbiome, has been shown to activate the host immune system, promoting the production of RNase7 by keratinocytes, thereby preventing pathogen colonization on the host skin [36]. This extensive evidence links the skin microbiome closely to innate immune responses, indicating its critical role in maintaining skin health and protecting the body from infections.

#### Adaptive immunity

The skin microbiome is also essential for adaptive immune responses. During the early life establishment of the immune system in newborns, interactions between skin commensal microorganisms and dendritic cells induce the formation of commensal‐specific regulatory T (Treg) cells in the host, establishing immune tolerance [37].

During the early developmental stages of infants, the development of natural killer T cells and mucosal‐associated invariant T cells is closely related to the metabolic products of the skin microbiome [38]. Regulatory T cells are specialized T lymphocytes in the immune system, primarily responsible for regulating immune responses and maintaining immune balance and self‐tolerance. In mouse models, the skin commensal *S. epidermidis* can directly induce the production of many regulatory T cells within a specific time window during the neonatal period, particularly within the first to second week after birth. This process is crucial for establishing immune tolerance and skin homeostasis [38]. The long‐term presence of Tregs in the skin determines the host's response to commensal microorganisms in adulthood. When Tregs in the skin exhibit specific adaptive defects, specific T cells may produce abnormal type 2 cytokines, potentially leading to several inflammatory skin diseases [39]. Overall, multiple lines of evidence indicate the critical role of the skin microbiome in regulating the host's adaptive immune system.

## 3 THE SKIN MICROBIOME AND DISEASE

**FIGURE 2:**
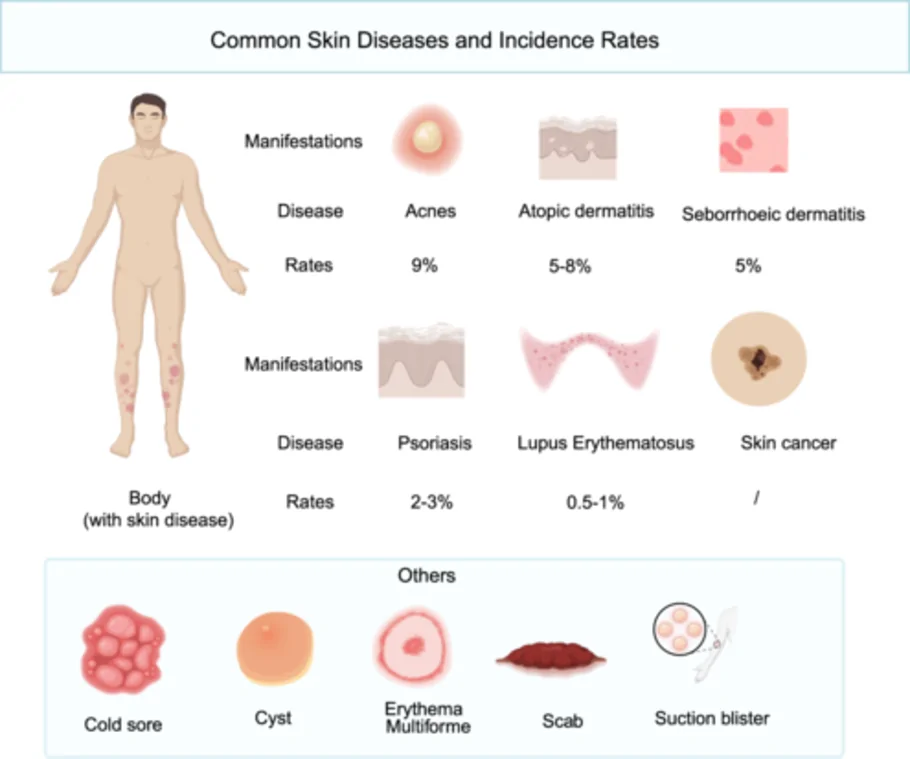
Common skin diseases, incidence rates, and symptoms. Inflammatory skin diseases have a high incidence rate, with acne being the most common skin disease. Similarly, autoimmune diseases such as psoriasis and lupus erythematosus also have high incidence rates. The incidence rate of skin cancer differs from other skin diseases in statistical methods.

Skin diseases affect the lives of millions of people worldwide. They pose significant threats to the physical and mental health of patients, severely impacting their quality of life. Additionally, skin diseases often require long‐term treatment and management, significantly increasing the burden on societal healthcare resources. According to the International Classification of Diseases, 10th Revision (ICD‐10), over 1000 skin or skin‐related diseases are listed, but a few common skin diseases account for most skin disease cases [40] (Table 1). Although it remains unclear for many skin diseases whether changes in the skin microbiome are a cause or a consequence of the disease, numerous studies have demonstrated a close association between specific skin microorganisms and certain skin diseases (Figure 2). Here, we review the close relationships between some of the most common skin diseases and the skin microbiome, providing potential targets for microbiome‐based therapies.

**TABLE 1:** The association between skin diseases and skin microbiome.

Disease	Skin microbiome/microorganism	Reference
Warts	Cutaneous warts are common skin lesions, caused by the Human Papillomavirus (HPV).	[57]
Herpes zoster	Herpes zoster is highly associated with Varicella‐zoster virus (VZV).	[41]
Tinea infections	Tinea infections are mostly caused by fungi of the genus *Trichophyton*, *Microsporum*, and *Epidermophyton*.	[42]
Atopic dermatitis	*Staphylococcus aureus* secretes virulence factors such as superantigens, enzymes, and other proteins.	[43]
	Cellular cytokines associated with *Malassezia* exacerbate inflammatory reactions.	[44, 45]
Acne	*Cutibacterium acnes* subsp*. acnes* (type I)	[46, 47]
Seborrheic dermatitis	*Malassezia. restricta* and *Malassezia. globosa* are the most common among patients.	[48]
	There is a significant positive correlation between the intensity of *Malassezia* growth and the severity of symptoms of SD.	[49]
Psoriasis	Changes in the structure and function of the skin microbiome.	[50]
Lupus erythematosus	*Staphylococcus aureus* promotes SLE and similar autoimmune inflammatory responses.	[51]
	The skin microbiome in SLE‐affected rash areas shows decreased alpha and beta diversity compared to healthy skin.	[52]
Melanoma	The relative abundance of *Streptococcus* and *Staphylococcus* significantly increased.	[53]
	The prevalence of *Corynebacterium* is higher in late‐stage patients.	[54]
Cutaneous squamous cell carcinoma	The abundance of *Staphylococcus* aureus increased, while the abundance of *Cutibacterium acnes* and *Malassezia* decreased.	[55]
Merkel cell carcinoma	Viral infections contribute substantially to the global cancer burden, with Merkel cell polyomavirus (MCPyV) being the most recently identified human oncogenic virus.	[56]

### Infectious skin diseases

Infectious skin diseases are caused by pathogens such as bacteria, viruses, fungi, or parasites and can spread through direct human contact, contact with contaminated objects, or the environment. These diseases are typically highly contagious and include common conditions like impetigo, scabies, herpes zoster, and ringworm, posing potential threats to public health and individual well‐being.

#### Warts

Cutaneous warts are common skin lesions caused by the Human Papillomavirus (HPV). In most cases, these lesions resolve on their own within months of the initial infection, with or without treatment. The infection is most prevalent during the second decade of life, affecting more than 40% of children [57]. Warts appear in various forms on different sites of the body and include common warts (verruca vulgaris), plane or flat warts, myrmecia, plantar warts, coalesced mosaic warts, filiform warts, periungual warts, anogenital warts (venereal or condyloma acuminata), oral warts and respiratory papillomas [58].

According to the online PaVe database (https://pave.niaid.nih.gov/), there are currently over 400 known types of human papillomaviruses (HPVs). These types are grouped into five genera: α, β, γ, µ, and ν [59]. It is transmitted through contact with HPV particles. Epidemiological data on cutaneous wart‐associated HPV types are rare. HPV 27, 57, 2, and 1 are the most prevalent HPV types in cutaneous warts in general population [60].

#### Herpes zoster

Herpes zoster causes significant suffering owing to acute and chronic pain or postherpetic neuralgia. Varicella‐zoster virus‐induced neuronal destruction and inflammation cause the principal problems of pain, interference with activities of daily living, and reduced quality of life in older adults [41]. Varicella‐zoster virus (VZV), a neurotropic alpha herpesvirus, causes chickenpox and shingles and is found worldwide [61]. HZ occurs globally without seasonal fluctuations in incidence. The incidence of HZ is age‐dependent, ranging from 1.2 to 3.4 cases per 1000 individuals per year in younger adults, to 3.9 to 11.8 cases per 1000 individuals per year in older adults (aged >65 years) [62].

VZV is found worldwide, though it is more prevalent in temperate climates. Primary VZV infection triggers the production of IgG, IgM, and IgA antibodies, which bind to various viral proteins. Effective virus‐specific cellular immunity is essential for controlling VZV replication, both in healthy individuals and in those who are immunocompromised, during primary or recurrent infections [63].

#### Tinea infections

Tinea infections are fungal skin conditions caused by dermatophytes, categorized by the affected site, including tinea corporis (body), tinea capitis (scalp), tinea cruris (groin), tinea pedis (feet), and tinea unguium (nails, also called onychomycosis) [42]. These infections present varied symptoms, often mimicking conditions like eczema, alopecia areata, or nail dystrophy, making clinical diagnosis challenging.

Tinea infections affect about 25% of the world population, and the filamentous fungus Trichophyton rubrum is the main causative agent of this group of diseases [64]. Tinea infections are mostly caused by fungi of the genus *Trichophyton*, *Microsporum*, or *Epidermophyton* [42]. Cutaneous manifestations of tinea infections are seen worldwide and classified based on the affected body site [42]. In North America, the cause is almost exclusively *Trichophyton tonsurans* [65].

### Inflammatory skin diseases

Inflammatory skin diseases are characterized by local inflammation of the skin tissues, often caused by various factors. Here, we focus on the role of the skin microbiome in inflammatory skin diseases.

#### Atopic dermatitis

Atopic dermatitis (AD) is a common chronic inflammatory skin disease affecting 11%–20% of children and 5%–8% of adults worldwide [66]. Clinically, it is characterized by erythema, scaling, and itchy lesions, which are prone to recurrence, significantly impacting the quality of life of patients, their families, and caregivers [67, 68]. AD is a multifaceted condition marked by a complex interplay between genetic predispositions, environmental triggers, and host–microbiome interactions. An overgrowth of *S. aureus* is frequently observed in moderate to severe AD, often correlating with heightened inflammation and disease severity [69]. *S. epidermidis* may become dominant in certain patients with milder disease. *S. epidermidis* is generally considered a commensal organism that can contribute to skin barrier function and immune homeostasis, partly by producing antimicrobial peptides that inhibit other potential pathogens [27, 70, 71]. However, its role in AD remains nuanced, as it can also elicit inflammatory responses under specific conditions [72]. This variation in microbial dominance among AD patients underscores the heterogeneous nature of the disease, wherein different microbial shifts may reflect or influence distinct clinical presentations and severities. Understanding these microbial dynamics is crucial for developing targeted microbiome‐based interventions and personalized treatment strategies.

The skin microbiome participates in AD‐related inflammatory responses through multiple pathways. *S. aureus* colonizes the skin of AD patients, secreting virulence factors such as superantigens, enzymes, and other proteins, leading to skin barrier dysfunction and delivering allergens to epidermal dendritic cells, thereby exacerbating AD symptoms [43]. Additionally, the extensive colonization of pathogenic *S. aureus* may reduce the abundance of other commensal microorganisms capable of producing antimicrobial peptides, which could contribute to skin barrier dysfunction and may exacerbate AD symptoms [73]. Genetic factors are also associated with *S. aureus* colonization; for instance, loss‐of‐function mutations in the *FLG* gene result in abnormal keratinocyte morphology, making them more susceptible to *S. aureus* binding and colonization, thus increasing the risk of AD [74]. Studies have found that some common skin commensals, such as species of *Cutibacterium*, can promote *S. aureus* colonization by accelerating its aggregation and biofilm formation [75]. Furthermore, *Malassezia* species, normally commensal on healthy skin, can exacerbate AD inflammation through the IL‐23/IL‐17 pathway [44]. *Malassezia sympodialis* can release extracellular vesicles that induce IL‐4 and TNF‐α cytokines, aggravating AD‐related inflammation [45]. KR Chng et al. observed a significant decrease in the relative abundance of *Streptococcus, Gemella spp., and Dermacoccus spp*. and increased ammonia production capacity in AD patients through metagenomic analysis [76].

#### Acne

Acne is also a common chronic inflammatory skin disease, affecting approximately 9% of the global population and being the most prevalent skin condition in the United States [77]. The incidence peaks between ages 15–25, characterized by comedones, inflammatory papules, pustules, and nodulocystic lesions, often leading to long‐term sequelae, including scarring and hyperpigmentation [78]. The pathophysiology of acne involves four main factors: (1) altered sebum production, (2) inflammation, (3) hyperkeratinization, and (4) *C. acnes* [79]. The complex pathogenesis of acne involves the skin barrier, genetics, and the immune system. Dysbiosis of the skin microbiome, particularly the overgrowth and imbalance of *C. acnes* subtypes, is crucial in acne development [46, 80, 81, 82].

*C. acnes* is a Gram‐positive facultative anaerobe on human skin, closely associated with acne [83]. It is divided into three subspecies: *C. acnes* subsp. *acnes* (type I), *C. acnes* subsp. *defendens* (type II), and *C. acnes* subsp. *elongatum* (type III). While type I, particularly subtype IA, is most closely associated with acne [46, 47], type II is linked to skin infections and prostate cancer [84], and type III is associated with progressive macular hypomelanosis [85]. Comparative proteomics of *C. acnes* subspecies suggests that type I induces inflammatory cytokines IFN‐γ and IL‐17, potentially promoting acne by activating Th1 and Th17 responses [86]. All *C. acnes* strains can produce CAMP factors, membrane‐perforating toxins with cytotoxic potential for keratinocytes and macrophages, potentially causing skin inflammation [87]. Porphyrins, secreted by *C. acnes*, absorb light under UV and visible spectra, contributing to follicular inflammation in acne [82]. Subtype IA1 strains isolated from acne patients produce higher levels of porphyrins than type II strains from healthy skin, possibly explaining the inflammatory response in acne [88].

#### Seborrheic dermatitis

Seborrheic dermatitis (SD) is a chronic, recurrent inflammatory skin condition characterized by erythematous plaques with varying degrees of scaling and associated pruritus, occurring in seborrheic areas such as the scalp, face, chest, back, axillae, and groin [89]. The global prevalence of SD is approximately 5%, with males being three times more likely to be affected than females, and incidence increasing with age [90]. Although the pathogenesis of SD involves host sebaceous gland activity, immunosuppression, endocrine factors, and neurological factors, the interaction between the skin microbiota (especially *Malassezia*) and skin surface lipids plays a central role [91]. The abundant colonization of *Malassezia* fungi on the skin is a key characteristic of seborrheic dermatitis [92].

*Malassezia* species (formerly *Pityrosporum*) are lipophilic yeasts constituting a significant part of the healthy skin microbiome, comprising 17 species. All *Malassezia* species exhibit high lipase activity, with *Malassezia globosa* having the highest, producing 14 lipases. These enzymes hydrolyze sebum triglycerides, generating fatty acids that support *Malassezia* growth and enhance its pathogenicity. *M. restricta* and *M. globosa* are the most common species associated with SD [48]. *Malassezia* species also produce reactive oxygen species, inducing cytotoxic lipid peroxidation and damaging the skin barrier [93]. Moreover, *Malassezia* can convert tryptophan into indoles, ligands for the aryl hydrocarbon receptor (AhR), affecting immune responses in cells expressing AhR [94]. Another study indicated that dsRNA from *Malassezia* stimulates Toll‐like receptor 3‐mediated immune responses associated with SD [95]. Recent research involving 168 patients and 30 healthy volunteers showed a significant positive correlation between *Malassezia* growth intensity and SD symptom severity, highlighting *Malassezia*'s role in SD pathogenesi [49].

### Immune diseases

#### Psoriasis

Psoriasis is an immune‐mediated chronic skin disease. As of the early 2020s, psoriasis is estimated to be 2%–3%, affecting approximately 60 million people worldwide, with an average onset age of 33 years [96]. The incidence is similar between males and females. Psoriasis is characterized by red, scaly patches that are often itchy and can lead to significant psychological distress, with up to 20% of patients experiencing depression. The disease substantially burdens individuals and society due to its physical and mental impact [97, 98].

Genetic studies at the familial and population levels have demonstrated that heredity is a significant factor in the development of psoriasis, with heritability estimates ranging from 60% to 90% [99]. The pathogenesis of psoriasis is primarily related to the body's adaptive immune response. Still, the expression of psoriasis‐related genes is influenced by environmental factors such as stress, alcohol consumption, and smoking. Changes in the structure and function of the skin microbiome also play a critical role in the disease process [50].

Numerous studies have shown significant dysbiosis in the skin microbiome of psoriasis patients [100]. In a study involving 75 patients and 124 healthy volunteers, Alekseyenko et al. observed that alpha diversity and beta diversity were significantly reduced in the skin microbiome of psoriasis patients [101]. Another study by Drago L et al. collected swabs from 54 psoriasis patients and 37 healthy controls, finding that the skin microbiome of psoriasis patients was characterized by reduced taxonomic diversity and a significant increase in the relative abundance of *Proteobacteria* [102].

Fyhrquist et al. analyzed the skin microbiome in a larger cohort, including individuals with AD, psoriasis, and healthy volunteers, discovering that a decrease in the abundance of *Corynebacterium* might play a regulatory role in psoriasis [103]. The exact mechanisms by which the skin microbiome contributes to psoriasis pathogenesis remain to be further explored and confirmed. Most researchers believe that the skin microbiome's role in psoriasis is primarily related to regulating the host's adaptive immune system. One crucial pathway involves the skin microbiome stimulating the host to produce cytokines such as LL‐37, TNF‐α, and iNOS, which induce T cells to differentiate into Th17 cells, leading to the production of interleukin‐17 (IL‐17) and interleukin‐22 (IL‐22), thereby promoting psoriasis development [103]. As our understanding of psoriasis pathogenesis deepens, the metabolic processes and therapeutic targets involving the skin microbiome will become clearer.

#### Lupus erythematosus

Lupus erythematosus (LE) is a clinically heterogeneous autoimmune disease that can manifest as a purely cutaneous condition or as part of systemic lupus erythematosus (SLE). LE affects 0.5%–1% of the global population. Approximately 80% of SLE patients experience skin‐related symptoms during the disease [104]. SLE occurs in both males and females, but it is more prevalent in females, with a female‐to‐male ratio of about 6:1 and an average age of onset around 30 years [105].

The etiology of SLE involves a combination of genetic and environmental factors, including epigenetic variations such as DNA methylation or histone modifications that lead to dysregulated gene expression and activation of innate and adaptive immunity [106]. Recently, the role of the skin microbiome in SLE has garnered increasing attention. Changes in the structure and function of the skin microbiome in SLE patients suggest a potential link between microbiome dysbiosis and SLE pathogenesis, presenting possible biomarkers and therapeutic targets for future treatment [107].

Most studies indicate that the skin microbiome in SLE‐affected rash areas shows decreased alpha and beta diversity compared to healthy skin. Cancan Huang et al. found that SLE patients had decreased skin community richness and evenness compared to healthy controls, with greater heterogeneity [52]. Huang et al. also found that differences in the skin microbiome between SLE patients and healthy individuals primarily involved *S. aureus* and *S. epidermidis* [52]. Zhou et al. similarly observed reduced diversity and richness in the rash areas of SLE patients, with *Halomonas* being more prevalent in SLE rash areas, while *Novosphingobium, Curvibacter, and Pelagibacterium* were less abundant in non‐rash areas of SLE patients [51]. Recent studies have also mechanistically linked *S. aureus* to SLE pathogenesis. Hitoshi Terui et al. discovered that *S. aureus* colonizing the skin can induce keratinocyte apoptosis and neutrophil activation through a caspase‐mediated process, activating dendritic cells and T cells to induce an IL‐23/IL‐17 immune response, promoting SLE and similar autoimmune inflammatory responses [108].

Although the exact etiology and mechanisms of SLE are not yet fully understood, advancements in next‐generation sequencing and whole metagenomic shotgun sequencing have significantly improved our understanding of the role of the skin microbiome in SLE pathogenesis. Increasing evidence suggests that the microbiome plays a crucial role in developing SLE [104].

### Cancer

#### Melanoma

Among various skin cancers, melanoma (MM) is not the most common, but it is potentially the most lethal. Statistics show that deaths related to malignant melanoma account for 75% of all skin cancer deaths [109]. Melanoma most commonly affects individuals aged 40–60. Annually, melanoma results in approximately 55,500 deaths, and for over four decades, there have been few available treatment options and clinical trials [110].

Recently, the gut microbiome has been recognized as a potential new player in the pathogenesis and treatment of malignant melanoma [111]. Some studies have applied gut microbiome‐based treatments for melanoma, such as engineered probiotics and fecal microbiota transplantation [112, 113]. The role of the skin microbiome in melanoma is also gradually being revealed. Pigs are considered the best model species for skin research due to their skin's structural similarity to human skin. Jarosz ŁS et al. collected 90 samples to study melanoma‐associated skin microbiome changes in a study using pigs as model animals. The results showed that the relative abundances of *Streptococcus* and *Staphylococcus* were significantly increased in melanoma samples compared to healthy skin microbiome samples [53].

A study by Mizuhashi et al. found that the prevalence of *Corynebacterium* was significantly higher in stage III/IV melanoma patients (76.9%) compared to stage I/II patients (28.6%). Additionally, more IL‐17‐positive cells were detected in Corynebacterium‐positive patients than in Corynebacterium‐negative patients [54]. Previous studies have shown that IL‐17 can promote melanoma cell proliferation by upregulating IL‐6 and signal transducer and activator of transcription 3 [114]. A recent study also confirmed dysbiosis of the skin and gut microbiomes in melanoma progression. In a pig model study, *Fusobacterium*, *Trueperella*, *Staphylococcus*, *Streptococcus*, and *Bacteroides* were significantly more abundant in the melanoma tissue microbiome compared to healthy controls [115]. Notably, Nakatsuji et al. found that intravenous injection of 6‐HAP from *S. epidermidis* could inhibit the growth of the B16F10 melanoma cell line, suggesting a potential protective role of *S. epidermidis* against MM [116].

Future research needs to continue exploring the role and mechanisms of the skin microbiome in melanoma pathogenesis, developing potential skin microbiome‐based therapies to improve melanoma patients' survival.

#### Cutaneous squamous cell carcinoma (cSCC)

As a complex epithelial cancer, cSCC is driven by a combination of germline and somatic genetic and epigenetic changes, environmental interactions, and immune evasion [117]. cSCC originates from epidermal keratinocytes and is the second most common epithelial malignancy, often clinically presenting as indurated, crusted lesions [118]. cSCC is one of the most common cancers among Caucasians, accounting for nearly one‐third of all non‐melanoma skin cancers (NMSC) and 20% of all skin tumors, with an annual incidence of over one million cases in the United States [119]. The lifetime risk of cSCC is estimated to be 6%–11%, and this incidence continues to increase [120].

Most squamous cell carcinomas develop from precancerous actinic keratosis (AK) lesions formed on sun‐exposed skin. Although the role of the skin microbiome in this process is not yet fully understood, some evidence suggests a connection. David L. A. Wood et al. longitudinally analyzed the microbiome of 112 AK lesions and conducted a cross‐sectional analysis of 32 spontaneously appearing SCC lesions. They found that the relative abundances of *Cutibacterium* and *Malassezia* strains, commonly present on non‐lesional skin, were significantly higher than in lesional skin; conversely, *S. aureus* strains were more abundant in lesional skin [55].

Another recent study by Anita Y Voigt et al. involving 81 patients diagnosed with cSCC and AK, and 25 healthy volunteers found similar results. The study showed that multiple *Lactobacillus* species, such as *Lactobacillus rhamnosus* and *Lactobacillus plantarum*, were less abundant in AK and SCC, while Ralstonia, particularly R. pickettii, were more abundant in AK and SCC. Notably, the skin commensal *C. acnes* was significantly reduced in AK and SCC patients, accompanied by an increase in the pathogenic *S. aureus* [121]. Annika Krueger et al. recently discovered that *S. aureus* strains isolated from cSCC skin cancer secrete toxins such as IL‐6, IL‐8, and TNF‐α, promoting tumor inflammation in patients' skin, which may explain the increased abundance of *S. aureus* in cSCC patients [122].

Further exploration of the changes and roles of the skin microbiome in the pathogenesis of cSCC is needed, aiming to develop new skin microbiome‐based adjunctive therapies and provide more clinical treatment options for patients.

#### Merkel cell carcinoma

Merkel cell carcinoma (MCC) is a rare and aggressive form of skin cancer, often linked to viral infections or sun exposure, which strongly activates the immune system [123]. Merkel cell carcinoma occurs at a rate of approximately 0.7 cases per 100,000 person‐years in the United States [124].

Viral infections contribute substantially to the global cancer burden, with Merkel cell polyomavirus (MCPyV) being the most recently identified human oncogenic virus [56]. Merkel cell polyomavirus, the first polyomavirus proven to be directly associated with human cancer, was recently discovered, shedding light on many of the puzzling characteristics of Merkel cell carcinoma [125]. While in vitro and in vivo models have offered valuable insights into the molecular functions of viral oncoproteins in cellular transformation, numerous aspects of the virus's natural life cycle, mechanisms of persistence, and the specific role of MCPyV in MCC pathogenesis remain unclear [126].

## 4 THE SKIN MICROBIOME ENGINEERING

**FIGURE 3:**
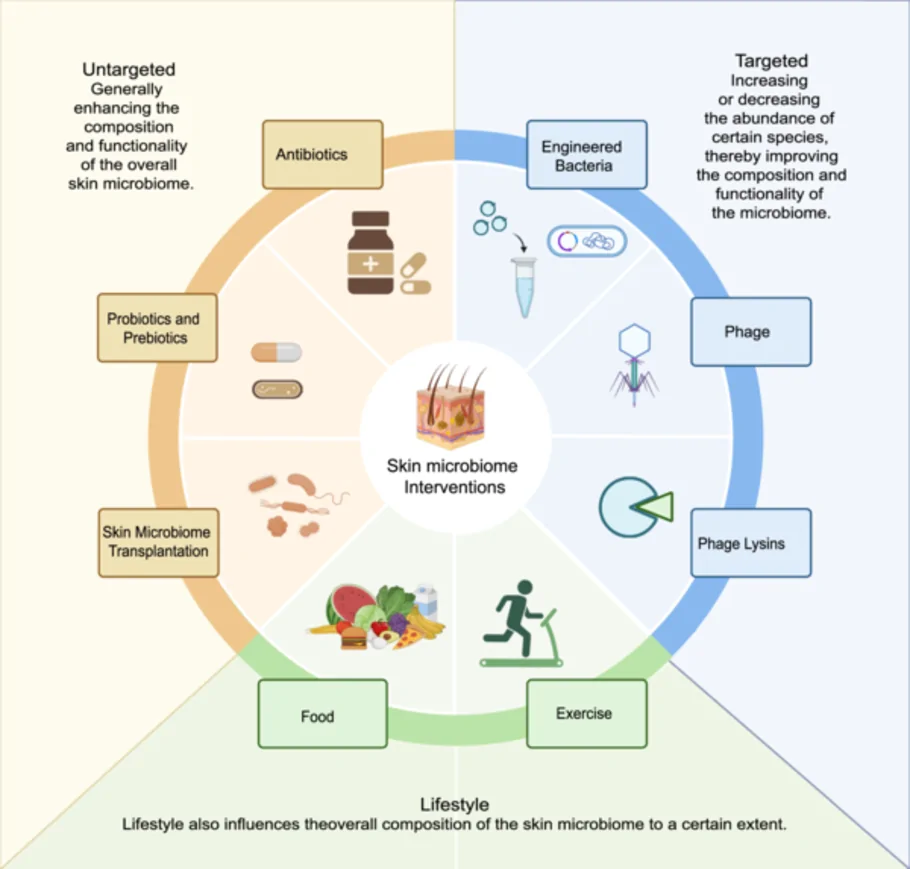
Strategies for regulating the skin microbiome. Nontargeted interventions modulate the overall composition of the skin microbiome, while targeted approaches selectively eliminate specific pathogens or potential pathogenic microorganisms or alter the abundance and functionality of particular microbial taxa, resulting in significant shifts in microbiome structure and function. Additionally, lifestyle factors and other interventions, such as skin care routines, dietary habits, and physical or chemical treatments, influence the skin microbiome by shaping its ecological balance and microbial diversity.

As discussed above, the skin microbiome plays a critical role in skin diseases. In this section, we will focus on various methods of regulating the human skin microbiome to treat skin diseases and improve skin health (Figure 3), including both the untargeted and targeted approaches (Table 2).

**TABLE 2:** The current skin diseases therapy type.

Therapy type	Specific method	Current status	Suitable skin conditions	Advantages	Disadvantages	Future developments and solutions
Nontargeted	Antibiotics	Mature technology, widely used in clinical treatments; well‐studied with clear side effects and contraindications; however, high research and development costs, long cycles, and low market returns limit new antibiotic development.	Various bacterial infections such as wound infections, and acne.	Broad‐spectrum action can quickly reduce multiple pathogens; effective for acute bacterial infections; cost‐effective and mature technology.	Imbalance of skin microbiome, affecting beneficial bacteria; severe resistance issues, leading to worse infections.	Development of new narrow‐spectrum antibiotics to minimize the impact on beneficial bacteria; integration with probiotics to restore skin microbiome; use of AI and deep learning to accelerate new antibiotic discovery; government incentives to stimulate antibiotic research and development investments.
	Skin Microbiome Transplant	Early developmental stage, mainly focused on research and preliminary applications.	Acne, atopic dermatitis, psoriasis.	Improves diversity and health of skin microbiome, promotes skin barrier repair; relatively simple operation; low cost.	Limited donor availability, potential pathogen transmission; uncertain long‐term stability; complex standardization and ethical approval processes; difficulty in long‐term colonization; potential immune risks.	Development of synthetic microbiomes to reduce reliance on donors; establishment of standardized procedures to enhance safety and efficacy.
	Probiotics and Prebiotics	Introduced into various skincare products; lacking systematic clinical studies to support efficacy.	Acne, atopic dermatitis, seborrheic dermatitis.	High safety, suitable for long‐term skin health management; enhances immune response, reduces inflammation; promotes skin barrier function, such as improving hydration and reducing allergic reactions.	Efficacy varies among individuals; stability and activity of probiotics require careful control; prebiotics may lack specificity and be utilized by various microbes; insufficient persuasive clinical evidence.	Large‐scale randomized controlled trials to validate efficacy; development of personalized probiotics for specific individuals or diseases; improvement of prebiotic specificity for targeted actions.
Targeted	Engineered Bacteria	Experimental stage, with a few successful cases; still in the early phase without widespread application.	Acne, atopic dermatitis, wound infections.	Precise modulation of microbiome functions for targeted regulation; personalized design for specific patients to produce antimicrobial substances or metabolites.	Safety and long‐term effects need verification; potential unpredictable interactions with host skin.	Development of safer engineered bacteria to minimize genetic escape risks; optimization of production processes to reduce costs; integration of synthetic biology tools to enhance safety and controllability.
	Phage Therapy	Still in the early phase without broad adoption, extensive clinical research mainly targeting antibiotic‐resistant bacterial infections.	Acne, Multi‐drug‐resistant *Staphylococcus aureus* (MRSA) infections, skin ulcers.	High specificity, selectively eliminates pathogenic bacteria without affecting other commensal bacteria; effective for multidrug‐resistant bacteria (e.g., MRSA).	Requires precise pathogen identification and diagnostics; narrow host range; risk of phage resistance development; inconsistent global regulatory policies.	Genetic engineering to expand phage host range, enhance their bactericidal capabilities, and reduce the likelihood of resistance; development of rapid diagnostic techniques to enhance targeting; strengthened ethical review and public education to reduce ethical disputes.
	Phage Lysin	Some products are in the initial stages with a promising market; most are still in preclinical research.	Acne, MRSA infections, atopic dermatitis.	High efficiency, specificity with low resistance risks; can be optimized via genetic engineering; effective against resistant bacteria.	High production costs; delivery challenges in vivo; lack of long‐term safety validation; insufficient clinical cases.	Improvement of production processes to lower costs; development of more effective delivery systems; extensive clinical trials to validate effectiveness and safety in treating skin diseases.

### Untargeted Approaches

#### Antibiotics

Antibiotics are hailed as one of the greatest medical discoveries of the 20th century, significantly reducing morbidity and mortality caused by microbial infections. The period from the 1940s to the 1960s is known as the “golden age” of antibiotic discovery, during which numerous antibiotics were developed and produced by pharmaceutical companies. However, due to the low‐profit margins of traditional antibiotics, they have become less attractive to investors, resulting in few new antibiotic drugs in the development pipeline today [127]. Antibiotic resistance has become one of the most pressing challenges in modern medicine [128]. The CDC's 2021 report states that antibiotic‐resistant microorganisms cause over 2 million infections in the United States annually, resulting in more than 23,000 deaths. If no action is taken, it is estimated that by 2050, more than 10 million people will die from infections caused by antibiotic‐resistant bacteria [129].

Despite the development of many new drugs and treatments for skin diseases over the past few decades, antibiotics remain the first choice for many skin conditions due to their broad‐spectrum mechanisms of action, including (i) inhibiting bacterial cell wall biosynthesis, (ii) disrupting cell membrane integrity, (iii) inhibiting nucleic acid or protein synthesis, and (iv) interfering with various metabolic processes [130]. For skin disease patients, antibiotics—whether injected, orally administered, or topically applied—inevitably disrupt the balance of the skin microbiome and can be toxic to human cells, potentially causing more severe diseases. These reasons have led to strict limitations on antibiotic use. In the “post‐antibiotic era,” developing new antibiotic alternatives has become a hot topic among researchers.

#### Skin microbiome transplantation (SMT)

Fecal microbiota transplantation (FMT) involves transplanting the microbiome from a healthy donor's feces into a patient's gut to improve the patient's gut microbiome structure and function [131]. This paradigm has been widely applied in treating gut microbiome‐related diseases in recent years. Similar to FMT, SMT involves replacing a patient's dysbiotic skin microbiome with a healthy skin microbiome, aiming to improve the physiological state of the affected skin through the complex interactions between the skin microbiome and the host. With a deeper and more comprehensive understanding of the skin microbiome's role in health and disease, the use cases for SMT are becoming clearer. Similar to FMT, the SMT process is being increasingly standardized and regulated [132, 133].

In recent years, SMT has been applied in some cases to treat skin diseases. Acne is the most prevalent skin disease worldwide. In a study by Paetzold et al., SMT was used to improve acne symptoms by transplanting a healthy skin microbiome (approximately 10^8 CFU/mL) onto the affected areas of patients for three consecutive days. The healthy skin microbiome was observed to colonize the patient's skin, with retention seen days later, indicating SMT's potential in treating skin diseases [134]. Another study by Chris Callewaert et al. explored using SMT to address body odor by replacing the dysbiotic skin microbiome in patients' armpits with a healthy skin microbiome [135]. Additionally, SMT has been proven feasible in treating skin diseases in companion animals. Kerem Ural et al. alleviated feline atopic syndrome (FASS) in cats through SMT, significantly reducing indices like the feline dermatitis extent and severity index and visual analog scale (VAS) pruritus score [136].

Despite the successes of SMT, most current studies use donor skin microbiomes directly sourced from healthy individuals, which poses various unavoidable issues, including ethical concerns, potential pathogens, and the scarcity of suitable donors. Researchers have begun exploring alternatives to traditional SMT. Benji Perin et al. attempted SMT between different body sites within the same individual to reduce pathogen risk and avoid ethical issues [137]. However, significant differences between skin microbiomes from different body sites, limited by the distinct physicochemical properties, restrict this approach's application.

In the future, synthetic consortia will be an excellent choice for SMT donors. Synthetic consortia refer to microbiomes designed and constructed to perform specific biological functions or produce particular chemicals. Compared to natural microbiomes, synthetic consortia offer more advantages in microbiome transplantation, including achieving specific functions, reducing potential risks, and facilitating standardization and regulation. This makes synthetic consortia a potentially safer, more precise, and personalized SMT strategy. However, challenges remain, including potential safety risks and long‐term impacts of synthetic consortia in human disease treatment, which need to be effectively addressed and validated before clinical application. However, ensuring the long‐term colonization of these engineered microbial communities remains a critical challenge. While synthetic consortia can be tailored to specific therapeutic needs and standardized more easily than natural donors, their sustained presence and functional stability on the skin over extended periods are yet to be fully validated. Future research efforts should focus on optimizing consortia design to promote robust colonization and interaction with the host, as well as establishing standardized assessment methods to monitor colonization persistence and clinical efficacy. By addressing these challenges, synthetic consortia hold the promise of becoming a safer, more precise, and personalized SMT strategy for long‐term disease management.

#### Probiotics and prebiotics

Probiotics are defined as live microorganisms that, when administered in adequate amounts, confer a health benefit on the host [138]. These benefits include improving the structure and function of the gut microbiome, enhancing host immunity, and lowering cholesterol levels [139]. The use of probiotics originated centuries ago when humans first noticed the health benefits of consuming fermented foods. Modern probiotic foods and products are direct derivatives of these early fermented foods. The concept of prebiotics builds on the concept of probiotics. In 1995, prebiotics were first defined as “a non‐digestible food ingredient that beneficially affects the host by selectively stimulating the growth and/or activity of one or a limited number of bacteria in the colon.” This concept and definition have evolved, and in 2016, the International Scientific Association for Probiotics and Prebiotics updated the definition of prebiotics to “a substrate that is selectively utilized by host microorganisms, conferring a health benefit [140].”

The pursuit of beauty is endless, and in recent years, the role of probiotics and prebiotics in various fields has been increasingly recognized. Their use in maintaining skin health is being extensively studied and discussed, including whitening, moisturizing, and antiaging applications. The role of probiotics and prebiotics in treating skin diseases, including AD, acne, and psoriasis, has also garnered widespread attention [141, 142, 143].

Topical probiotics have been shown to increase skin ceramide levels, improve erythema, scaling, and itching, and reduce the relative abundance of pathogenic *S. aureus* in atopic and seborrheic dermatitis. Studies have demonstrated the effectiveness of various probiotics, including *Streptococcus thermophilus*, *Vitreoscilla filiformis*, and *S. epidermidis* [144]. In a recent randomized, double‐blind trial involving infants with AD, *Lacticaseibacillus rhamnosus* showed significant benefits for the skin and gut, highlighting its potential for AD treatment [145]. Furthermore, a phase 1 randomized clinical trial with adults using *Staphylococcus hominis* A9 demonstrated its ability to reduce *S. aureus* colonization, inhibit inflammatory toxin expression, and improve local skin inflammation, underscoring the promising role of skin commensal bacteria in AD management [146].

Given probiotics' strong capabilities and potential in treating skin diseases, some probiotic drugs are already in development. Forte B‐401 is a topical medication composed of three therapeutic strains of the commensal Gram‐negative bacterium *Roseomonas mucosa*. In preclinical studies and Phase I/IIa clinical trials, FB‐401 demonstrated capabilities in promoting skin tissue repair, exhibiting anti‐inflammatory properties, and inhibiting potentially harmful bacteria like *S. aureus*. Unfortunately, according to the latest developments, it failed to show effective therapeutic outcomes in the Phase II clinical trial, leading Forte Biosciences to discontinue the development of this pipeline [147].

In the skincare field, probiotics and prebiotics have been widely used [148, 149]. Lactobacillus members are the most commonly used probiotics in skincare products. A recent study showed that *Lactobacillus plantarum*‐GMNL6 significantly reduced melanin synthesis in volunteers' skin while inhibiting the proliferation of *S. aureus* and *C. acnes*, indicating its potential for whitening and preventing pathogen colonization [150]. Another study found that a topical cream containing live *Lactobacillus reuteri* improved body odor by altering the armpit skin microbiome structure [151].

*Bifidobacterium* is one of the most widely studied probiotic genera in gut microbiome research and has a significant role in skincare. Recent research found that *Bifidobacterium animalis* subsp. *lactis* MG741 could prevent wrinkles and skin inflammation in cell experiments and mouse models by regulating skin photoaging markers, demonstrating its potential in sun protection [152]. Another study showed that Bifidobacterium could effectively reduce IL‐8 and TNF‐α secretion and COX‐2 mRNA expression in LPS‐induced THP‐1 macrophages, highlighting its role in enhancing skin barrier function and maintaining skin defense homeostasis [148].

Given the limitations of live bacteria use, some researchers have found that heat‐inactivated bacteria are also potential tools for regulating the skin microecosystem. Evidence suggests that heat‐inactivated Lactobacillus preparations can promote wound healing [153].

The extensive application of probiotics and prebiotics in skin care and skin disease treatment has demonstrated their vast potential. Although some potential human skin probiotics have been identified, many skin microbiota remain to be discovered and cultured. With advances in metagenomics analysis and high‐throughput sequencing technologies, we expect to find more probiotic and prebiotic resources in the future. Additionally, many probiotics used in skin care and skin disease treatments are based on gut microbiome research. However, the skin and gut environments are vastly different, and microorganisms not considered probiotics in the gut may have potential benefits for the skin. We need to explore the potential benefits of these microorganisms in the skin environment further and fully utilize their potential in skin care and treatment.

### Targeted approaches

#### Engineered bacteria

Engineered bacteria represent a crucial method in the engineering of the skin microbiome. Specifically, by modifying certain microorganisms, these bacteria can effectively contribute to maintaining skin health or be utilized to treat skin diseases. Engineered bacteria achieve precise gene editing typically using the CRISPR‐Cas system, a highly efficient tool for cutting, inserting, or modifying specific DNA sequences in the genome, offering significant potential for disease treatment. Researchers are exploring using engineered bacteria to improve skin health, treat skin diseases, and achieve personalized medicine, providing new potential therapeutic strategies for future skin microbiome engineering and showcasing the possibilities for personalized medical approaches to skin diseases.

*C. acnes*, the most common skin commensal, is considered the most attractive chassis organism for skin delivery therapy. Still, it has long lacked effective gene editing methods due to its difficulty in editing. Recently, a study successfully used gene editing technology to make *C. acnes* produce and secrete a therapeutic molecule, Neutrophil Gelatinase‐associated Lipocalin (NGAL), in mice [154]. NGAL is a protein shown to help alleviate acne symptoms by inducing sebocyte apoptosis [155]. This study provides essential evidence for the potential use of engineered skin commensal *C. acnes* in treating acne.

Although relatively few cases of engineered bacteria are being used to treat skin diseases, engineered bacteria therapy is already a common method in the gut microbiome field. In a recent study, researchers engineered a series of yeast strains with different butyrate synthesis capacities to meet different patients' needs. These engineered yeast strains regulated the gut microbiome and butyrate content in the gut, significantly improving colitis in a mouse model [156]. *E. coli* is a model organism for gene editing. Researchers used gene editing on the probiotic strain *E. coli Nissle* 1917 as a host, showing in vivo prevention and treatment activity against *Pseudomonas aeruginosa* during gut infection in two animal models (*C. elegans* and mice) [157]. *Vibrio cholerae* (*V. cholerae*) is the culprit behind the severe intestinal infectious disease cholera. In another study, researchers developed an in vitro sensing and killing system in wild‐type *E. coli* to target the pathogenic *V. cholerae*. The designed *E. coli* specifically detected *V. cholerae* through its quorum‐sensing molecule CAI‐1 and responded by expressing the lytic protein YebF‐Art‐085, self‐lysing, and releasing the killing protein Art‐085 to kill *V. cholerae* [158].

The significant achievements of engineered bacteria in the gut microbiome field suggest similar potential in skin microbiome regulation and treatment. This indicates that engineered bacteria may have more applications and breakthroughs in the future, bringing new possibilities for skin microbiome intervention and treatment.

The application of engineered bacteria in the skin microbiome field faces several challenges. Firstly, safety concerns involve potential safety hazards and the uncertainty of long‐term effects. Additionally, the standardization and scaling of engineered bacteria production and the validation of their effectiveness in clinical practice are technical challenges. Future research needs to focus on feasible editing methods for engineered bacteria to meet skin disease treatment needs. Another limitation is the lack of a suitable animal model for studying the skin microbiome. Furthermore, the long‐term safety and effectiveness of engineered bacteria for skin disease treatment need to be deeply studied, advancing the standardization and regulation of engineered bacteria therapy to ensure treatment consistency and effectiveness. Additionally, exploring the application of engineered bacteria in preventive treatments for skin diseases can help maintain skin microbiome balance and health, preventing the occurrence and development of skin diseases.

#### Phage therapy

Phages, or bacteriophages, are viruses that infect bacteria. Their lifecycle involves infection, replication, and release. In phage therapy, these viruses are used as therapeutic agents to target and destroy bacterial infections. Phage therapy was widely used in Eastern Europe and the former Soviet Union, but it wasn't until the 21st century that the standardization and regulation of phage therapy began to take shape [159]. The specificity of phages allows them to selectively eliminate specific bacteria in certain scenarios without affecting other host commensal microorganisms, thus avoiding harm to host cells. For the skin microbiome, phage therapy can target pathogenic microorganisms without disrupting the skin's microbial ecology and barrier function. However, this specificity is also a disadvantage as it requires accurate diagnosis and identification of the pathogen, sometimes down to the strain level, which can be time‐consuming and resource‐intensive, making it difficult to achieve in clinical settings in a short time [160].

Another significant advantage of phage therapy is its effectiveness against multi‐drug‐resistant (MDR) bacteria. Antibiotic resistance is one of the greatest threats to global health today, and phage therapy is seen as a viable alternative to antibiotic treatments. Additionally, due to the genetic diversity of phages and their widespread distribution in the environment, the potential sources of phages in nature are nearly limitless, providing a vast pool of candidates for phage therapy [161].

Phage therapy has been successfully applied to treat various infections. Recently, Rebekah M Dedrick et al. reported a case of phage therapy used to treat antibiotic‐resistant *Mycobacterium abscessus* infections. On a compassionate‐use basis, phages were administered via intravenous injection or nebulization to 20 selected patients, with 11 showing favorable clinical or microbiological responses [162].

There is also evidence suggesting the potential of phage therapy in treating skin diseases. As discussed in Part 2, *C. acnes* is closely associated with acne. Recently, Min‐Hui Han et al. reported a new *C. acnes* phage, PAP 1‐1. In vitro experiments showed that combining this phage with Nisin could specifically kill *C. acnes* KCTC 3314 without affecting other skin commensal microorganisms, demonstrating its potential in acne treatment [163]. Amit Rimon et al. recently demonstrated the targeted clearance of *C. acnes* using phage therapy in a mouse model. The study found that combining *C. acnes* phages with antibiotics eradicated 100% of *C. acnes* strains in the mouse acne model and reduced inflammation‐related cytokines, significantly lowering skin inflammation, highlighting the potential of phage therapy in acne treatment [164].

Multi‐drug‐resistant *Staphylococcus aureus* (MRSA) skin infections have long plagued doctors and patients, and phage therapy is seen as a hope for treating skin and soft tissue infections caused by MRSA. Many successful cases have been reported in recent years. Recently, Zhvania P et al. reported a case of compassionate use of phage cocktail therapy for a patient with Netherton syndrome who was allergic to multiple antibiotics, resulting in significant clinical improvement within 7 days and a substantial improvement in quality of life after 6 months of continuous phage cocktail treatment [165]. Phage therapy has also shown efficacy in treating skin infections caused by antibiotic‐resistant *Pseudomonas aeruginosa* and *Mycobacterium marinum*. These pieces of evidence suggest the significant potential of phage therapy in treating skin infections [166, 167].

However, natural phages also have several drawbacks, including bacterial resistance to natural phages, the difficulty of applying lysogenic phages in phage therapy, and the off‐target effects of natural phages. With a deeper understanding of phage structure and mechanisms, the superiority of engineered phages is gradually emerging. Dedrick, R.M. et al. reported converting two lysogenic phages into lytic phages and combining them with other lytic phages to form a phage cocktail to combat MDR *Mycobacterium abscessus* infections. *Mycobacterium abscessus* can cause various lung, skin, and soft tissue diseases, central nervous system, and ocular infections. Their study screened over 10,000 isolated mycobacteriophages to find suitable lytic phages for *Mycobacterium abscessus*, discovering that only Muddy was a lytic phage. The other two (ZoeJ and BPs) were lysogenic phages but were engineered to become lytic, thus making them suitable for use in a designed phage mixture to treat MDR *Mycobacterium abscessus* infections [168].

Moreover, the long‐term safety of natural phages still needs further verification. Engineering phages through various modifications can address three main issues of natural phages: (1) targeting specificity, (2) bacterial lysis efficiency, and (3) safety. Engineered phage therapy has already been applied in treating several diseases. In a recent study, the CRISPR‐Cas system was used to engineer phages to selectively kill *E. coli*, causing lethal infections in leukemia patients with better efficiency than wild‐type phages [169]. Although phages are unlikely to completely replace antibiotics in treating skin diseases, phage therapy can serve as an effective supplement for patients and doctors. However, the safety of phage therapy has always been a limitation to its widespread use.

#### Phage lysins

Enzymes are the center of biochemical reactions, with exceptional catalytic functions. Since their discovery, enzymes have been widely applied across various industries. Over the past decades, enzyme therapy has also rapidly developed and is used to treat multiple diseases, including lysosomal storage diseases, cancer, and Alzheimer's disease. Due to the widespread presence of antibiotic‐resistance genes, enzymes are considered effective antibiotic alternatives to combat diseases caused by resistant bacteria, directly killing or inhibiting bacterial growth.

Phage lysins are among the most intensively studied antibiotic alternative enzymes. Phage lysins are characterized by high specificity, efficiency, and low resistance risk, making them one of the best antibiotic alternatives for treating MDR bacteria. Phage lysins are a type of peptidoglycan hydrolase that can cleave essential covalent bonds in the bacterial cell wall, disrupting its structure and leading to cell lysis. Studies have shown structural domain differences in phage lysins targeting Gram‐positive and Gram‐negative bacteria. Researchers have developed potential phage lysins for treating skin diseases.

Ja‐I Kim recently reported a phage lysin isolated from the *C. acnes* phage CAP 10‐3. The lysin showed significant antibacterial activity against *C. acnes* in vitro through heterologous expression, indicating its potential as an effective acne treatment [170]. However, natural phage lysins have several issues, including (1) poor stability, (2) weak targeting, and (3) low lysis efficiency. With advancements in synthetic biology and materials science, engineering phage lysins is becoming a hot research topic to enhance or alter specific properties. Some engineered phage lysins have shown exceptional properties and have been successfully applied in some cases. Fritz Eichenseher et al. discovered an effective *S. aureus* phage lysin, which was improved through recombination and modification at domain interfaces. The engineered lysin showed significantly enhanced lysis efficiency and effectively targeted and cleared *S. aureus* in a mouse model. Fusing antimicrobial peptides (AMPs) to both ends of phage lysins is another effective engineering modification. In a study by Christina Varotsou et al., two short AMPs were fused to the N‐terminus of lysins from the *C. acnes* phage PAC1, improving lytic activity against *C. acnes*, *Enterococcus faecalis* and *Enterococcus faecium* [171].

Several phage lysin drugs are currently in clinical validation. CF‐301, or Exebacase, is a lysin undergoing phase III clinical trials for treating Gram‐positive bacterial infections, particularly MRSA. However, its clinical response in phase III did not show the desired effect and has been discontinued [172]. SAL200 also exhibits broad‐spectrum bactericidal activity against *S. aureus* isolates and is currently in phase IIa clinical trials [173]. Extensive evidence suggests the significant potential of phage lysins, and with further understanding of their mechanisms, structures, and production, phage lysins may play a more prominent role in the skin microbiome field.

Phage lysins, as potential antibacterial treatments, face several limitations and challenges. The production cost of phage lysins is a critical factor limiting their application, as medical use requires high purity, activity, and stability, increasing the technical difficulty of large‐scale production [174]. Additionally, stringent quality control requirements for medical‐grade phage lysins raise production costs. Overcoming these limitations and challenges requires further research and technological innovation to achieve broader clinical applications of phage lysins. Future research should focus on enhancing lysin specificity, stability improvements, cost reduction, and exploring innovative application methods to develop superior phage lysins and expand their application scenarios.

### Lifestyle

Lifestyle factors significantly impact the composition of the skin microbiome, and these effects are often long‐lasting and filled with uncertainties. Compared to targeted and nontargeted microbiome therapies, lifestyle factors exert broader and more complex influences. Factors such as diet, exercise, skincare products, and medications, among others, all shape the ecological balance of the skin microbiome to varying degrees.

Specifically, dietary habits are well‐known to correlate closely with the composition of the gut microbiome and, to some extent, influence the composition and structure of the skin microbiome. Some studies have reported that the gut–skin axis is important in human health [175].

Moderate exercise can enhance the body's immune defenses, help maintain the integrity of the skin barrier, and prevent the invasion of pathogenic microorganisms while providing a stable environment for beneficial microbes on the skin. A robust immune system is more effective at identifying and eliminating pathogenic microorganisms, thereby supporting a healthy balance within the skin microbiome. The choice of skin care products is critical to the health of the skin microbiome, as certain potent chemical ingredients can disrupt the micro‐ecological balance of the skin, leading to the proliferation of harmful bacteria or a reduction in beneficial ones. In contrast, gentle and hydrating skincare products can help maintain the skin's normal pH level, enhance the skin barrier function, and support a healthy microbial environment. These products provide adequate moisture and nutrients, minimize disruption to the microbiome, promote microbial diversity and stability, and contribute to overall skin health [176]. Notably, some studies have found that work and study‐related stress can influence the human skin microbiome and the severity of skin conditions, particularly showing a positive correlation between acne severity and perceived stress levels [177, 178]. Other factors also significantly impact the human skin microbiome, which has been comprehensively summarized in another review [179].

Due to individual differences, the effects of these factors can vary significantly among different populations. In the treatment of certain skin diseases, improving these factors can help alleviate symptoms to some extent and support therapeutic outcomes. As researchers gain a deeper understanding of how environmental factors influence the composition of the skin microbiome, there is potential to leverage these easily modifiable lifestyle aspects better to assist in the treatment of skin disorders in the future.

### Others

In some specific cases, other physical and chemical methods may serve as supplements or alternatives to the microbiome modulation methods mentioned above. This section primarily introduces laser therapy, cryotherapy, and gas therapy.

#### Cryotherapy

Cryotherapy, commonly referred to as cold therapy, is a clinical procedure that uses extremely low temperatures, primarily liquid nitrogen, to address various skin conditions. This method is highly effective against warts caused by the human papillomavirus, skin tags, and some types of NMSC. The process involves rapidly freezing the targeted skin tissue with liquid nitrogen, which is briefly applied to the skin. The intense cold causes the cells within the tissue to crystallize and die, eliminating unwanted or abnormal skin tissues in the process.

This therapy has been applied in the treatment of many skin diseases. A study demonstrated the potential of cryotherapy in acne treatment, showing that precise cryotherapy can effectively improve acne lesions [180]. Cryotherapy has also played a significant role in reducing the burden of specific pathogens, particularly those directly causing skin lesions. For instance, in the treatment of viral warts, the application of liquid nitrogen effectively destroys viral particles within the warts, thereby reducing the risk of reinfection [181]. Additionally, numerous skin cancer treatments are closely associated with cryotherapy [182].

Overall, while the effects of cryotherapy on the skin microbiome are generally localized and temporary, its ability to reduce pathogenic burden can contribute to a healthier microbial balance, particularly in areas affected by direct pathogenic invasion. Ongoing research and clinical monitoring are recommended to better understand the long‐term implications of cryotherapy on skin health and microbial dynamics.

#### Laser therapy

Laser therapy is a versatile dermatological treatment that uses focused light of specific wavelengths to target various skin issues, ranging from cosmetic concerns to medical conditions. This method is highly effective for treating skin discoloration, scars, vascular lesions, and even certain types of skin cancer [183].

Photobiomodulation (PBM) is a newly adopted consensus term that replaces the therapeutic application of low‐level laser therapy. PBM can play a role in modulating the abundance of specific bacterial species, particularly *Staphylococcus*, a significant pathogenic strain on the skin [184]. Laser therapy has been widely applied in the treatment of various skin cancers, with numerous documented cases [185, 186].

The impact of laser therapy on the skin's healing process can also indirectly influence the microbiome's reestablishment. The new cellular environment and the skin's altered immune response posttreatment create conditions conducive to microbial growth and colonization.

Although the primary focus of laser therapy is its physical effects on skin tissues, its influence on the skin microbiome, though localized and generally mild, underscores the importance of further research to fully understand the implications of laser treatments on skin health and microbial dynamics. Such understanding is crucial for optimizing posttreatment care and improving long‐term treatment efficacy, ensuring that patients receive the most benefit from this advanced therapeutic technology.

#### Gas therapy

Gas therapy is an emerging dermatological treatment that utilizes therapeutic gases such as ozone, nitric oxide, or carbon dioxide to address skin‐related microbial imbalances and promote wound healing. By delivering active gas molecules to the affected areas, gas therapy can inhibit the growth of pathogenic microorganisms, including drug‐resistant strains such as *S. aureus* [187].

The impact of gas therapy extends beyond its antimicrobial properties, as it promotes skin barrier recovery and strengthens local immune defenses, fostering a healthier environment for microbial growth. This dual effect is particularly valuable in treating conditions characterized by microbial imbalances and inflammation, such as acne or chronic wounds. Additionally, gas therapy's selective antimicrobial action makes it a promising alternative to traditional antibiotics, minimizing the risk of resistance development while maintaining a more favorable balance in the skin microbiome. These treatments have shown some progress in the treatment of certain types of skin cancer [188].

While the primary focus of gas therapy is its physiological effects on skin tissues, its influence on the skin microbiome highlights the importance of further research to understand its broader implications for skin health and microbial dynamics. This understanding is essential for optimizing treatment protocols and ensuring that patients benefit from this innovative and minimally invasive therapeutic approach.

## 5 DISCUSSION

Early sequencing technologies and traditional culturing methods have provided us with a basic understanding of the skin microbiome, and most past research has been based on these techniques and strategies. With the development of next‐generation amplicon sequencing and metagenomic sequencing technologies, our understanding of the skin microbiome has become more profound [189]. However, these new technologies are not without their drawbacks. Because skin samples are typically swabs that contain tiny amounts of bacteria and high amounts of host DNA, there is a need for higher sample processing and analysis capabilities [190]. The lack of clear consensus and standards for skin microbiome sampling has been a significant reason for conflicting research results. Future research efforts should aim to establish standardized sampling guidelines and consensus.

Although our understanding of the skin microbiome no longer relies solely on traditional culturing methods, we have only successfully cultured a small fraction of skin microorganisms to date [191]. There is no doubt that future research should continue exploring methods for culturing previously unculturable skin microorganisms to expand our knowledge and understanding of the skin microbiome.

Furthermore, the causality between skin microorganisms and skin diseases remains unclear for most associations, with only a few potential pathogenic microorganisms and their mechanisms being deeply studied. Although pigs are considered the best animal models for skin disease, conducting pig‐based skin disease model experiments poses challenges for most research, and significant differences still exist between pig skin and human skin structures and compositions [192]. Future research may need to focus on developing more suitable animal models for skin diseases to better understand the mechanisms and interactions between the skin microbiome and host skin diseases.

As more interaction mechanisms between skin microorganisms and skin diseases become clearer, they will provide more targets for skin microbiome engineering therapies [193]. With the rapid development of synthetic biology, more tools and technologies will be available for regulating and modifying the skin microbiome. Additionally, as the number of skin microbiome engineering therapy cases increases, there will be a need to clearly define and rigorously review the application scenarios for each therapeutic approach.

With the rapid iteration of generative artificial intelligence and deep learning, future researchers need to explore how to leverage increasingly mature AI to support traditional and emerging tools and technologies in skin microbiome engineering. This will require thoughtful consideration and exploration to harness AI's potential in advancing skin microbiome research and applications.

## AUTHOR CONTRIBUTIONS

**Yiang Lyu**: Writing—original draft; writing—review and editing; investigation; conceptualization. **Juntao Shen**: Writing—review and editing; project administration; methodology. **You Che**: Writing—review and editing. **Lei Dai**: Supervision; resources; project administration; writing—review and editing.

## CONFLICT OF INTEREST STATEMENT

The authors declare no conflicts of interest.

## ETHICS STATEMENT

No animals or humans were involved in this study.

## Data Availability

No new data and scripts were used for this review. Supplementary materials (graphical abstract, slides, videos, Chinese translated version, and update materials) can be found in the online DOI or iMeta Science http://www.imeta.science/imetaomics/.
